# Human Versus Artificial Intelligence: Comparing Cochrane Authors' and ChatGPT's Risk of Bias Assessments

**DOI:** 10.1002/cesm.70044

**Published:** 2025-08-31

**Authors:** Petek Eylul Taneri

**Affiliations:** ^1^ Cochrane Evidence Synthesis Unit Germany/UK, Institut für Allgemeinmedizin (ifam), Universitätsklinikum Düsseldorf Heinrich‐Heine‐Universität Düsseldorf Germany

**Keywords:** artificial intelligence, evidence synthesis, large language models, risk of bias

## Abstract

**Introduction:**

Systematic reviews and meta‐analyses synthesize randomized trial data to guide clinical decisions but require significant time and resources. Artificial intelligence (AI) offers a promising solution to streamline evidence synthesis, aiding study selection, data extraction, and risk of bias assessment. This study aims to evaluate the performance of ChatGPT‐4o in assessing the risk of bias in randomised controlled trials (RCTs) using the Risk of Bias 2 (RoB 2) tool, comparing its results with those conducted by human reviewers in Cochrane Reviews.

**Methods:**

A sample of Cochrane Reviews utilizing the RoB 2 tool was identified through the Cochrane Database of Systematic Reviews (CDSR). Protocols, qualitative systematic reviews, and reviews employing alternative risk of bias assessment tools were excluded. The study utilized ChatGPT‐4o to assess the risk of bias using a structured set of prompts corresponding to the RoB 2 domains. The agreement between ChatGPT‐4o and consensus‐based human reviewer assessments was evaluated using weighted kappa statistics. Additionally, accuracy, sensitivity, specificity, positive predictive value, and negative predictive value were calculated. All analyses were performed using R Studio (version 4.3.0).

**Results:**

A total of 42 Cochrane Reviews were screened, yielding a final sample of eight eligible reviews comprising 84 RCTs. The primary outcome of each included review was selected for risk of bias assessment. ChatGPT‐4o demonstrated moderate agreement with human reviewers for the overall risk of bias judgments (weighted kappa = 0.51, 95% CI: 0.36–0.66). Agreement varied across domains, ranging from fair (*κ* = 0.20 for selection of the reported results) to moderate (*κ* = 0.59 for measurement of outcomes). ChatGPT‐4o exhibited a sensitivity of 53% for identifying high‐risk studies and a specificity of 99% for classifying low‐risk studies.

**Conclusion:**

This study shows that ChatGPT‐4o can perform risk of bias assessments using RoB 2 with fair to moderate agreement with human reviewers. While AI‐assisted risk of bias assessment remains imperfect, advancements in prompt engineering and model refinement may enhance performance. Future research should explore standardised prompts and investigate interrater reliability among human reviewers to provide a more robust comparison.

## 1 Introduction

Systematic reviews and meta‐analyses synthesise data from randomised controlled trials (RCTs) to inform clinical decisions. However, they are increasingly time‐consuming and resource‐intensive, delaying access to crucial findings and impacting patient care [1, 2].

Artificial intelligence (AI) offers a potential solution by streamlining tasks such as search refinement, study screening, data extraction, and summarization [3]. While AI‐driven abstract screening has matured, data extraction remains a challenge, requiring further refinement in machine‐learning approaches [4].

Advancements in large language models (LLMs) like GPT‐4, Claude, and LLaMA2 have enhanced automation in evidence synthesis [5]. These models, trained on extensive text corpora, can perform complex tasks using natural language prompts, aiding systematic review processes, including study selection and risk of bias assessment [3, 6].

The Risk of Bias 2 (RoB 2) tool, introduced in 2019, is the standard for assessing bias in RCTs. While it provides structured judgements across five domains, its application remains labour‐intensive, often requiring over 2 h per study [7, 8]. AI tools like ChatGPT‐4 could potentially reduce this workload and improve efficiency.

This study aims to evaluate ChatGPT‐4o's performance in RoB 2 assessments, comparing its results with human evaluations in Cochrane Reviews.

## 2 Methods

Eligible Cochrane systematic reviews were identified from the Cochrane Database of Systematic Reviews (CDSR) using the keyword “systematic review” (7 December 2024 and 20 December 2024). An initial screening of the first 25 studies, sorted by newest to oldest publication date, was conducted. However, as most of these reviews used the RoB 1 tool and did not provide sufficient evaluations using the RoB 2 tool, the screening was extended to the first 50 studies to ensure an adequate number of randomised controlled trial assessments using RoB 2 were identified. Protocols, qualitative reviews, prognostic reviews, and diagnostic test accuracy reviews and those using non‐RoB 2 bias tools were excluded, while intervention reviews were included. Reviews focusing on traditional or complementary medicine were excluded due to variability in reporting quality and trial design, which may hinder consistent application of the RoB 2 tool and limit comparability with other intervention reviews. RCTs reporting both dichotomous and continuous outcomes and assessed using RoB 2 were included.

As RoB 2 is an outcome‐based tool, assessments were conducted at the outcome level rather than for entire studies. The primary outcome reported in each included Cochrane review was used as the basis for both the Cochrane reviewers' and ChatGPT's risk of bias judgments.

No formal sample size calculation was performed. The selection of reviews was based on feasibility considerations, as all assessments were conducted by a single researcher. Therefore, the final sample size reflects a balance between methodological rigour and practical constraints.

The prompt was developed based on Lai et al.'s approach, which involved senior experts establishing criteria from guidelines [9], then adapted for relevance to the RoB 2 tool by incorporating insights on improving ChatGPT prompts [10, 11]. and further refined using information from RoB 2 developers, including the “crib sheet summarising the tool” [12].

ChatGPT‐4o was queried using structured prompts based on RoB 2's signalling questions (January 2025). Responses included risk of bias ratings (low, some concerns, high) and the predicted direction of bias. A final prompt summarised ChatGPT's assessments for consistency. Full prompt and data collection form with the collected data is shared in the Open Science Framework (OSF) [13].

Data were collected in Microsoft Excel, with ChatGPT assessments performed sequentially by the author. Agreement between ChatGPT and Cochrane reviewers was measured using weighted kappa statistics [14]. Results of the kappa statistics were interpreted using standard guidelines: 0.0–0.2 (slight), 0.21–0.40 (fair), 0.41–0.60 (moderate), 0.61–0.80 (substantial), and 0.81–1.0 (perfect) agreement [15]. Accuracy, sensitivity, specificity, positive predictive value, and negative predictive value were calculated using a 3 × 3 confusion matrix. Accuracy was defined as the proportion of correct classifications made by ChatGPT compared to Cochrane authors' judgments. While accuracy reflects overall agreement, kappa statistics adjust for agreement that may occur by chance. Statistical analysis was conducted in R Studio (version 4.3.0) using the “psych” and “caret” packages.

To account for potential clustering of RCTs within Cochrane reviews, a cumulative link mixed model was conducted with ChatGPT's risk of bias judgments as the outcome, Cochrane judgments as the predictor, and Cochrane review ID included as a random intercept.

## 3 Results

The majority of Cochrane systematic reviews (29 studies) were excluded from this analysis due to the use of the older Cochrane risk of bias tool. Eight Cochrane reviews met the eligibility criteria, yielding 84 RCTs. The data set, including exclusions, is available on the OSF [13].

The RCTs included in this analysis were published between 1993 and 2021. The majority of studies (60 out of 84, 71.4%) were derived from a single Cochrane Review examining the effects of physical exercise for individuals with Parkinson's disease [16]. Other studies addressed antibiotic use for leptospirosis [17], sympathetic nerve blocks for cancer pain [18], loop diuretics for acute heart failure [19], alcohol reduction during pregnancy [20], uterotonics for retained placenta [21], transcutaneous bilirubinometry [22], and SARS‐CoV‐2 workplace interventions [23].

**Graph 1:**
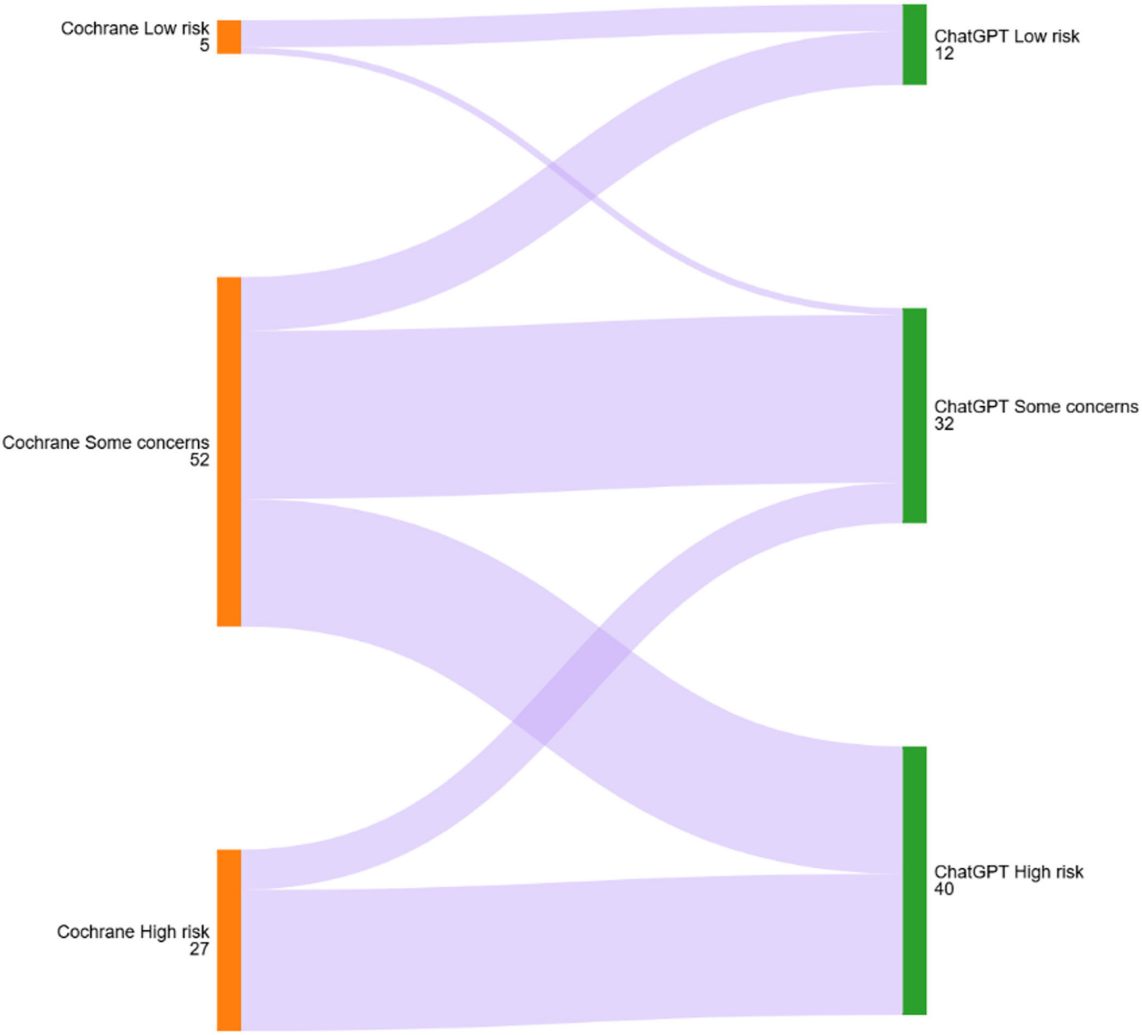
Comparison of ChatGPT and Cochrane authors' risk of bias assessments for overall bias.

ChatGPT‐4o's RoB judgments are summarised in Table 1. It did not misclassify studies with low risk as high risk or vice versa but assigned 36.5% (19/52) of studies rated as “some concerns” by Cochrane authors as high risk (Graph 1).

**Table 1:** Comparison of ChatGPT and Cochrane authors' risk of bias assessments (domain based and overall).

	ChatGPT																		
		Domain 1			Domain 2			Domain 3			Domain 4			Domain 5			Overall RoB		
Cochrane reviews		Low risk	Some concerns	High risk	Low risk	Some concerns	High risk	Low risk	Some concerns	High risk	Low risk	Some concerns	High risk	Low risk	Some concerns	High risk	Low risk	Some concerns	High risk
	Low risk	24	1	0	18	2	3	37	8	3	40	0	1	30	3	0	4	1	0
	Some concerns	15	38	2	24	18	16	4	3	1	22	1	11	28	5	1	8	25	19
	High risk	0	4	0	2	0	1	13	12	3	1	0	8	9	7	1	0	6	21

The weighted kappa for overall RoB was 0.51 (95% CI: 0.36–0.66), indicating moderate agreement. Agreement varied across domains, with the lowest kappa for domain assessing bias in the selection of the reported results (*κ* = 0.20, fair) and the highest for the domain assessing bias in the measurement of outcomes (*κ* = 0.59, moderate). Overall accuracy was 0.59 (95% CI: 0.48–0.70), with the highest domain‐specific accuracy for bias from randomisation (0.73, 95% CI: 0.63 to 0.82) and the lowest for selection of reported results (0.42, 95% CI: 0.32 to 0.54) (Table 2).

**Table 2:** Domain‐based and overall risk of bias assessment accuracy and Cohen's kappa statistics.

	Accuracy (95% CI)	Cohen's kappa (95% CI)
D1 (“randomization”)	0.73 (0.63, 0.82)	0.57 (0.44, 0.70)
D2 (“deviations from interventions”)	0.44 (0.33, 0.55)	0.21 (0.027, 0.39)
D3 (“missing data”)	0.51 (0.40, 0.62)	0.22 (0.04, 0.41)
D4 (“outcome measurement”)	0.58 (0.47, 0.69)	0.59 (0.44, 0.74)
D5 (“selective reporting”)	0.42 (0.32, 0.54)	0.20 (0.06, 0.34)
Overall RoB	0.59 (0.48, 0.70)	0.51 (0.36, 0.66)

ChatGPT‐4o's sensitivity for detecting high‐risk studies was 53%, with a specificity of 99% for identifying low‐risk studies (Table 3). Domain‐specific sensitivity ranged from 0% to 50%, while specificity ranged from 63% to 98% (Supporting Table 1).

**Table 3:** Overall risk of bias assessments' sensitivity, specificity, positive predictive value and negative predictive value statistics.

	Low risk of bias	Some concerns	High risk of bias
Sensitivity	0.33	0.78	0.53
Specificity	0.99	0.48	0.86
Pos pred value	0.80	0.48	0.78
Neg pred value	0.90	0.78	0.67

For Domain 1, the cumulative link mixed model adjusting for Cochrane review clustering showed negligible between‐review variance (3.07 × 10⁻⁹), and fixed effect estimates could not be interpreted due to undefined standard errors. Domain 2 demonstrated moderate between‐review variance (1.04) with a positive but nonsignificant association between Cochrane and ChatGPT judgments (*p* = 0.06). In Domain 3, moderate variance (0.58) was observed, with no significant association (*p* = 0.23). Domain 4 showed moderate variance (2.11) and a statistically significant positive association (*p* < 0.01). For Domain 5, between‐review variance was negligible (5.63 × 10⁻⁹), and convergence issues prevented interpretation of fixed effects. Finally, for the overall risk of bias judgment, the model indicated negligible variance (1.16 × 10⁻⁹); although it converged, standard errors and *p* values could not be estimated due to limited variability, restricting interpretability.

## 4 Discussion

This study assessed the level of agreement between RoB assessments conducted by Cochrane systematic review authors using the RoB 2 tool and those generated by ChatGPT‐4o for the same trials. The findings revealed that ChatGPT‐4o exhibited moderate agreement in overall judgments, while domain‐specific assessments varied from fair to moderate agreement. These results suggest a slightly higher level of concordance compared to previous studies. Given that effective prompt engineering can enhance the performance of ChatGPT [24], the development and validation of standardised prompts for risk of bias assessment are warranted. This would necessitate collaborative efforts between evidence synthesis researchers and AI specialists in prompt design.

To date, only few studies have assessed ChatGPT's performance in RoB assessments. One study employed a distinct methodological approach by differentiating between two modelling techniques: direct prediction of RoB 2 judgments and a decomposition method, wherein ChatGPT responded to a series of signalling questions before making an overall judgment. This study reported slight to fair agreement between ChatGPT and human reviewers [25]. A study conducted by Kuitunen et al. more closely resembled the methodology of the present research, although it used a single prompt, whereas the current study employed multiple prompts alongside a final prompt for re‐evaluation. Kuitunen et al. reported slight agreement in overall assessments, slight agreement in the bias due to randomisation domain, and agreement ranging from none to poor in other domains [26]. Pitre et al. tested ChatGPT's performance using three different prompts, with the “optimised prompt” yielding slight to fair agreement with Cochrane authors for both domain‐based and overall assessments [27]. Another study assessed Claude‐2, a widely used LLM, and found fair agreement for overall judgments and slight to fair agreement for domain‐specific assessments [6]. Further studies have explored the performance of LLMs using different risk of bias tools. Lai et al., whose prompt design informed the present study, evaluated ChatGPT and Claude with a modified Cochrane RoB tool and found both models demonstrated high accuracy, with Claude slightly outperforming ChatGPT (84.5% vs. 89.5%) [9]. Similarly, a study assessing ChatGPT 3.5 and 4 with the older Cochrane RoB tool found some agreement with human reviewers, though ChatGPT frequently provided indeterminate responses, underscoring issues related to consistency and precision [28]. A recent study evaluated ChatGPT's performance in risk‐of‐bias assessments of neonatal studies using the ROB 1 tool and found that the ChatGPT‐4o language model demonstrated overall weak agreement with Cochrane Neonatal Review risk‐of‐bias assessments, with similar discrepancies observed across most risk‐of‐bias domains [29]. Lastly, another study evaluated the accuracy and efficiency of GPT‐4o in applying the Jadad scale to assess risk of bias in randomised controlled trials in oral and maxillofacial surgery. Their findings indicated that GPT‐4o's assessments closely aligned with those of human reviewers, demonstrating high accuracy for overall scores (*κ* = 0.71–0.77). Moreover, the AI completed each assessment in approximately 15.2% of the time required by human reviewers, highlighting its potential for significantly improving efficiency.

In the present study, the lowest agreement was observed in the domain assessing bias in the selection of the reported results, whereas the highest agreement was found in the domain evaluating bias in the measurement of outcomes. These findings differ from previous research. Kuitunen et al. [26] reported the highest agreement in the bias due to randomisation domain and the lowest agreement in the bias due to selection of reported results. Similarly, Pitre et al. [27] found the lowest agreement in the domain assessing deviations from the intended intervention and the highest in the bias due to missing outcome data domain. Eisele‐Metzger et al. [6] also identified the lowest agreement in the domain assessing bias in the selection of the reported results but reported the highest agreement in the bias due to missing outcome data domain. Notably, Eisele‐Metzger et al. evaluated Claude rather than ChatGPT, which may contribute to the observed differences.

It is important to acknowledge that this study was conducted at a different time from previous studies, and LLMs evolve rapidly. Different versions of the same LLM may produce significantly different results. Among the studies reviewed, only Kuitunen et al. [26] used ChatGPT‐4o. Furthermore, the prompts used in previous research remain largely undisclosed, with the exception of Kuitunen et al., who applied a direct prompt that differed significantly from the multi‐prompt approach used in the present study. The use of stepwise prompting in this study aligns with expert recommendations for improving accuracy [30, 31].

While this study found limited agreement between Cochrane authors and ChatGPT‐4o in applying the RoB 2 tool, previous research has similarly documented suboptimal interrater reliability among human reviewers using the same tool. Minozzi et al. found that interrater reliability for overall judgments was slight, with domain‐based judgments ranging from slight to moderate agreement. Their study highlighted that, despite being a comprehensive tool, RoB 2 remains difficult to implement even for experienced systematic reviewers [32]. Similarly, Konsgen et al. observed variation in RoB 2 agreement across Cochrane reviews, with kappa values ranging from 0.19 (slight agreement) to 0.63 (substantial agreement) [33]. Additionally, Crocker et al. reported difficulties in implementing various aspects of the RoB 2 tool, despite extensive guidance. In addition to those challenges, Crocker et al. noted that the assessment process required an average of 5 h and 58 min of staff time per study [34]. Given these challenges, the limited uptake of RoB 2 among Cochrane authors is unsurprising; in this study, 28 Cochrane reviews had to be excluded due to reliance on older RoB tools. Investigating potential modifications to RoB 2 to enhance usability may be necessary to facilitate its implementation and improve reliability. Enhancing RoB 2's usability may also improve LLM performance in risk of bias assessments.

This study has several limitations. First, all assessments and analyses were conducted by a single researcher, without duplication to verify errors. Second, the possibility of low interrater reliability among Cochrane reviewers may have influenced agreement levels. A potential solution would be to conduct a randomised controlled study with experienced systematic reviewers to first establish interrater reliability before comparing human and LLM‐generated judgments. Additionally, the number of included reviews was limited due to the prevalence of older RoB tools in Cochrane systematic reviews, an issue requiring attention from Cochrane editors and moderators. Furthermore, 71% of the included RCTs originated from a single systematic review, which may have systematically influenced the results if interrater reliability within that review was low. However, the sensitivity analysis using cumulative link mixed models accounting for clustering by Cochrane review showed that agreement between Cochrane reviewers and ChatGPT varied by domain; a significant association was found only in Domain 4, while other domains demonstrated nonsignificant associations or were limited by negligible variance and convergence issues. A substantial proportion of the included RCTs were drawn from a single Cochrane review on physical exercise for Parkinson's disease, which may have introduced topic‐specific bias. Although a cumulative link mixed model was applied to account for clustering within reviews, this approach may not have fully addressed the influence of this dominant topic. The limited representation of diverse clinical areas, due in part to the scarcity of recent Cochrane reviews using the RoB 2 tool, further constrains the generalisability of the findings. Lastly, this study used static prompts only and did not evaluate interactive or adaptive prompting, which may further influence output quality and deserves future exploration.

In this study, ChatGPT assessed the risk of bias using only the information contained in the published study reports. Unlike human reviewers, the model was not provided with supporting materials such as trial protocols, registry entries, or related publications, which are often essential for a comprehensive RoB 2 assessment. This limitation reflects a practical constraint similar to that noted in another research [6], where Claude was evaluated without access to supporting documents. While human reviewers can consult multiple sources as needed, providing such materials to LLMs remains challenging due to their length and format, often requiring additional summarisation or prompt engineering. The absence of these additional inputs may have affected the completeness and accuracy of ChatGPT's judgments, and this limitation should be considered when interpreting the findings.

To aid interpretation of the findings, it is important to understand the roles of sensitivity and specificity in this context. High sensitivity for identifying high risk of bias judgments is crucial, as it ensures that studies with potential methodological flaws are not overlooked by the model. Conversely, high specificity is particularly relevant when identifying low risk of bias, helping to reduce false positives. As the reported sensitivity and specificity values refer to different risk categories, they are not directly comparable but should be interpreted within the context of their respective targets.

A key concern is the reproducibility of ChatGPT‐generated responses. Given that the same prompt may yield different outputs [35], and ChatGPT is continuously updated, future applications of this methodology could produce varying results, posing a challenge to scientific reproducibility. In addition, since some Cochrane Reviews are open access, ChatGPT may have been exposed to them during training. This could partially inflate performance and limit the generalisability of results to entirely new studies.

Furthermore, as with other large language models, ChatGPT‐4o relies exclusively on published content for training and output generation, which may limit its ability to fully capture contextual or unpublished nuances. These factors highlight the importance of prospective, real‐time evaluations to better understand and validate AI performance in this setting. In this study, ChatGPT was also prompted to provide explanations for its judgments, offering insight into the model's reasoning process. As AI tools advance, these explanations may serve as a reference for systematic reviewers, allowing human reviewers to function as “second reviewers” to verify ChatGPT's assessments. This could streamline systematic review processes and improve time management.

## 5 Conclusion

In conclusion, this study evaluated ChatGPT's utility in conducting RoB 2 assessments within Cochrane reviews, revealing fair to moderate agreement with Cochrane authors. While ChatGPT's performance remains suboptimal, it appears to demonstrate greater potential than in previous studies. Improving the design of prompts, especially in domains where agreement is low, may help increase the reliability of its assessments. Future research should prioritise establishing interrater reliability among human reviewers before directly comparing their assessments with LLM‐generated judgments. Future research should also focus on establishing interrater reliability among human reviewers before making direct comparisons with judgments produced by large language models. Since RoB 2 is typically applied through discussion and consensus among reviewers, it is important to acknowledge that tools like ChatGPT operate independently and do not benefit from this collaborative process. With this in mind, comparisons between AI and human assessments should be viewed as exploratory and interpreted within the context of these differences.

As AI continues to evolve, its potential to aid in systematic reviews warrants further exploration. As Louisa May Alcott wrote, “We should not be afraid of storms, for we are learning how to sail our ship.”

## Author Contributions

**Petek Eylul Taneri:** conceptualization, investigation, writing – original draft, methodology, visualization, writing – review and editing, software, formal analysis, data curation.

## Ethics Statement

The author has nothing to report.

## Consent

The author has nothing to report.

## Conflicts of Interest

The author declares no conflicts of interest.

## Peer Review

The peer review history for this article is available at https://www.webofscience.com/api/gateway/wos/peer-review/10.1002/cesm.70044.

## Supporting information


**Supplementary Table S1:** Domain 1‐5 sensitivity, specificity, PPV and NPV.

## Data Availability

The data that support the findings of this study are openly available in Open Science Framework at https://osf.io/z8thv/files/osfstorage/67a11002d00d4f55deb90160.
