# Supplementary material for: Human Versus Artificial Intelligence: Comparing Cochrane Authors' and ChatGPT's Risk of Bias Assessments
Source: Cochrane Evid Synth Methods. 2025 Aug 31;3(5):e70044. doi: 10.1002/cesm.70044 (PMC12407253; doi:10.1002/cesm.70044)
Supplement: Supplementary file 1 — Supplementary Table S1: Domain 1‐5 sensitivity, specificity, PPV and NPV. [file CESM-3-e70044-s001.docx]

|  | Domain 1 | | | Domain 2 | | | Domain 3 | | | Domain 4 | | | Domain 5 | | |
| --- | --- | --- | --- | --- | --- | --- | --- | --- | --- | --- | --- | --- | --- | --- | --- |
|  | Low risk | Some concerns | High risk | Low risk | Some concerns | High risk | Low risk | Some concerns | High risk | Low risk | Some concerns | High risk | Low risk | Some concerns | High risk |
| Sensitivity | 0.62 | 0.88 | 0.00 | 0.41 | 0.90 | 0.05 | 0.69 | 0.13 | 0.43 | 0.63 | 1.00 | 0.40 | 0.45 | 0.33 | 0.50 |
| Specificity | 0.98 | 0.59 | 0.95 | 0.86 | 0.38 | 0.97 | 0.63 | 0.92 | 0.68 | 0.95 | 0.60 | 0.98 | 0.82 | 0.58 | 0.80 |
| Pos Pred Value | 0.96 | 0.69 | 0.00 | 0.782 | 0.31 | 0.33 | 0.77 | 0.38 | 0.11 | 0.98 | 0.03 | 0.89 | 0.91 | 0.15 | 0.06 |
| Neg Pred Value | 0.75 | 0.83 | 0.98 | 0.57 | 0.92 | 0.77 | 0.53 | 0.74 | 0.93 | 0.47 | 1.00 | 0.84 | 0.27 | 0.80 | 0.99 |

Supplementary Table 1: Domain 1-5 sensitivity, specificity, PPV and NPV
